# Mesenteric SParIng *versus* extensive mesentereCtomY in primary ileocolic resection for ileocaecal Crohn’s disease (SPICY): study protocol for randomized controlled trial

**DOI:** 10.1093/bjsopen/zrab136

**Published:** 2022-02-15

**Authors:** E M L van der Does de Willebois, SPICY study group, W A Bemelman, C J Buskens, G R A M D'Haens, A D'Hoore, S Danese, M Duijvestein, K B Gecse, R Hompes, B Koot, F Indemans, A L Lightner, M W Mundt, A Spinelli, J D W van der Bilt, K W A van Dongen, S Vermeire, S Zwaveling

## Abstract

**Background:**

There is emerging evidence to suggest that Crohn’s disease (CD) may be a disease of the mesentery, rather than of the bowel alone. A more extensive mesenteric resection, removing an increased volume of mesentery and lymph nodes to prevent recurrence of CD, may improve clinical outcomes. This study aims to analyse whether more extensive ‘oncological’ mesenteric resection reduces the recurrence rate of CD.

**Methods:**

This is an international multicentre randomized controlled study, allocating patients to either group 1—mesenteric sparing ileocolic resection (ICR), the current standard procedure for CD, or group 2—extensive mesenteric ICR, up to the level of the ileocolic trunk. To detect a clinically relevant difference of 25 per cent in endoscopic recurrence at 6 months, a total of 138 patients is required (including 10 per cent dropout). Patients aged over 16 with CD undergoing primary ICR are eligible. Primary outcome is 6-month postoperative endoscopic recurrence rate (modified Rutgeerts score of greater than or equal to i2b). Secondary outcomes are postoperative morbidity, clinical recurrence, quality of life, and the need for (re)starting immunosuppressive medication. For long-term results, patients will be followed up for up to 5 years to determine the reoperation rate for recurrence of disease at the anastomotic site.

**Conclusion:**

Analysing these two treatment strategies in a head-to-head comparison will allow an objective evaluation of the clinical relevance of extensive mesenteric resection in CD. If a clinical benefit can be demonstrated, this could result in changes to guidelines which currently recommend close bowel resection.

**Registration number:**

NCT00287612 (http://www.clinicaltrials.gov)

## Teaser

There is emerging evidence to suggest that Crohn’s disease (CD) may be a disease of the mesentery, rather than of the bowel alone. It is hypothesized that patients undergoing a more extensive oncological mesenteric resection during an ileocolic resection (ICR), compared with those undergoing a mesenteric sparing ICR, have decreased recurrence rates. This is an international multicentre randomized controlled study, wherein patients will be allocated to either group 1—mesenteric sparing ICR, the current standard procedure for CD, or group 2—extensive mesenteric ICR. The primary outcome is 6-month postoperative endoscopic recurrence rate (modified Rutgeerts score of greater than or equal to i2b). Analysing these two treatment strategies in a head-to-head comparison will allow an objective evaluation of the clinical relevance of extensive mesenteric resection in CD. If a clinical benefit can be demonstrated, this could result in changes to guidelines which currently recommend close bowel resection.

## Introduction

Despite an expanding medical armamentarium, up to 75 per cent of patients with Crohn’s disease (CD) need a surgical resection at some point during the course of their disease^1^. Well known indications are stricturing and penetrating disease. Recently, it has been demonstrated that surgical resection can also be beneficial to patients not responding to immunomodulator therapy. The LIR!C trial and its long-term results demonstrated that, in patients with limited ileocaecal disease, an ileocolic resection (ICR) is a favourable treatment option, resulting in better patient-reported quality of life (QoL) and less medical treatment, when compared with escalation treatment with tumour necrosis factor (TNF)-α blockers^2^^,^^3^.

Unfortunately, surgery for CD is not curative. The 6-month endoscopic recurrence rate is estimated to be up to 60 per cent^4^. Within 5 years postoperatively, clinical recurrence is observed in up to 50 per cent of patients^1^. A significant volume of research has been conducted in an attempt to determine how to prevent postoperative recurrence of CD following an ICR. Some studies focussed on the timing of resuming postoperative medications. Others looked at surgical techniques, including the type of anastomosis^5–8^. Other than the potential decreased recurrence with the Kono-S anastomosis^8^, no surgical techniques seem to alter the postoperative recurrence rate of CD following an ICR.

There is emerging evidence to suggest that CD may be a disease of the mesentery, rather than of the bowel alone^9–13^. However, the mesentery is typically left *in situ* during close bowel resection for CD^14^, unlike oncological resections for colorectal cancer. Recently, the first retrospective study on mesenteric resection, alongside ICR, in CD has been published, reporting surgical recurrence rates of 40 per cent in the ICR group without resection of the mesentery, compared with 3 per cent in the ICR group with resection of the mesentery^15^. However, data are difficult to interpret due to the difference in follow-up between the two groups. Prospective data from an RCT are lacking.

## Methods

### Objectives

The primary aim of this study is to prospectively determine the postoperative endoscopic recurrence rates following mesenteric sparing resection *versus* extensive mesenteric resection, when performing an ICR for CD. Endoscopic recurrence is generally considered as an objective and accurate parameter for disease activity, and it has been demonstrated that endoscopic recurrence almost always precedes clinical symptoms^16^.

Results of this study will enable the authors to provide a consensus on current surgical management.

### Study design

The Mesenteric SParIng *versus* extensive mesentereCtomY in primary ileocolic resection for ileocaecal Crohn’s disease (SPICY) trial is a multicentre RCT where participants will be randomized in a 1 : 1 ratio to either group 1—mesenteric sparing ICR, the current standard procedure for CD, or group 2—more extensive mesenteric ICR, up to the level of the ileocolic trunk. The trial will be conducted in Amsterdam University Medical Centre (AUMC) in Amsterdam, Flevo Hospital in Almere, and Maasziekenhuis Pantein in Beugen, the Netherlands; Humanitas Research Hospital in Milan, Italy; University Hospital Leuven in Leuven, Belgium; the Cleveland Clinic in Cleveland, Ohio, USA; and other centres interested in participating in the trial. The multinational participation in this study will greatly facilitate international implementation of the study results. Hospitals will be allowed to start patient accrual after local approval has been obtained.

### Primary and secondary outcomes

The primary outcome is the endoscopic recurrence rate at 6 months following ICR, defined as modified Rutgeerts score of greater than or equal to i2b, as determined by central reading.

Secondary outcomes are postoperative morbidity, clinical recurrence, QoL, and the need for (re)starting immunosuppressive medication postoperatively. For long-term results, patients will be followed up for up to 5 years to determine the reoperation rate for recurrence of disease at the anastomotic site.

### Study population

All eligible patients with CD undergoing primary ICR for terminal ileitis of ileocolic disease will be considered for inclusion. For eligibility to participate in this study, a subject must meet all of the following criteria: adults of either sex, aged 16 years or older; ileo(colic) disease with an indication for primary ileocaecal resection or ICR; subject on any concurrent therapies; terminal ileitis (L1 or L3 disease), previously confirmed by endoscopy, with a recent update (within the last 3 months) of imaging (for example, ultrasound, MRI, CT enterography) before confirmation of eligibility, preferably discussed by the multidisciplinary team; ability to comply with the study protocol; and competency and ability to provide written informed consent.

Concurrent perianal and/or further proximal disease are not exclusion criteria.

A potential subject who meets any of the following criteria will be excluded from participation in this study: inability to give informed consent; patients under the age of 16 years; patients undergoing repeat ICR; clinically significant medical conditions within 6 months before the operation (for example, myocardial infarction, active angina, congestive heart failure, or other conditions that would, in the opinion of the investigators, compromise the patient’s safety); history of cancer of less than 5 years which might influence the patient’s prognosis; emergent operation; pregnancy or breastfeeding; inability to comply with postoperative assessments, imaging and endoscopy; and Kono-S anastomosis.

### Ethical consideration

The trial will be conducted according to Good Clinical Practice guidelines and the principles of the Declaration of Helsinki (2013)^17^. This study is approved by the Medical Ethical Committee of the AMC. The protocol is registered by the Dutch Central Committee on Research Involving Human Subjects (NL61632.018.18).

### Informed consent procedure

Eligible patients with terminal ileitis referred for outpatient counselling for surgical resection will be screened for inclusion criteria at outpatient clinic visits. Patients meeting the inclusion criteria will be offered the opportunity to participate in the trial following multidisciplinary team discussion and will need to sign a written consent form if they would like to be enrolled.

### Randomization

Randomization of consented study participants to either mesenteric sparing ICR or extensive mesenteric ICR will be done using an online-based system for allocation concealment (Castor EDC). Allocation concealment will be ensured, as the service will not release the randomization code until the patient has signed for informed consent and has been recruited into the trial. Randomization will not be stratified. There is no blinding to treatment allocation for the treating surgeon. The treatment will be blinded for all other physicians (for example, gastroenterologists, central readers) and patients. A statistician blinded to treatment allocation will analyse the data.

### Study outline

#### Preoperative

Before surgery, all patients must have had an endoscopy to confirm terminal ileitis, and a recent update (within the last 3 months) of imaging before inclusion (for example, ultrasound, MRI, or CT enterography). The Crohn’s Disease Activity Index (CDAI) score will be determined, and the following Quality of Life (QoL) questionnaires administered: EuroQol-5 Dimension (EQ-5D) questionnaire, 36-Item Short Form Health Survey (SF-36), and Inflammatory Bowel Disease Questionnaire (IBDQ). Baseline characteristics will be collected.

#### Surgery

**Fig. 1:**
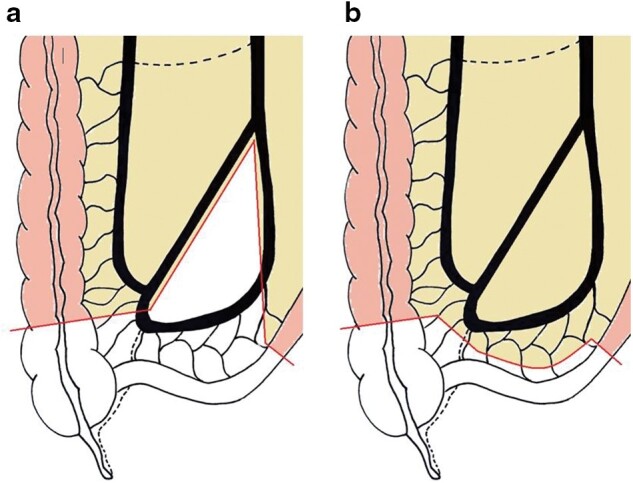
Surgical technique: a More extensive mesenteric resection following the lower border of the ileocolic trunk; b Mesenteric sparing ileocolic resection, conforming to current guidelines.

**Fig. 2:**
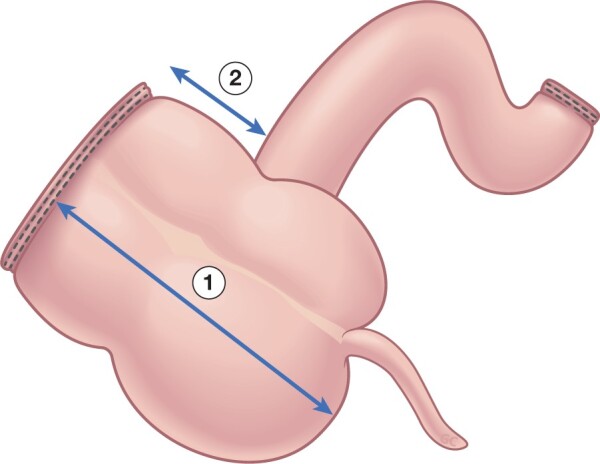
Measurement of length of resected specimen on colonic side 1, caecum and ascending colon; 2, ascending colon.

All surgical procedures will be performed as a laparoscopic resection, with conversion to an open operation only if clinically indicated. For mesenteric sparing resection, the mesentery is divided close to the bowel. In the more extensive mesenteric resection, the mesentery is resected up to the origin of the ileocolic trunk. After identification of the ileocolic pedicle, the lower border of the ileal branch of the ileocolic artery is followed distally until branching into caecal arteries. The rest of the operation will be identical. Care would be taken to ensure the amount of colon resected in both groups is similar (*Figs 1* and *2*). A wide-stapled anastomosis will be performed in all patients, as recommended by current European Crohn’s and Colitis Organisation (ECCO) guidelines. Technical aspects (for example, intra- or extracorporeal anastomosis) will be left at the discretion of the surgeon. The Kono-S anastomosis, and its role in the reduction of postoperative recurrence, is still a subject of research. Therefore, this technique will not be used in the current trial, as it can be considered a confounding variable. Operative data will be collected. Perioperatively, patients will be treated according to the local enhanced recovery pathway.

#### Quality control of surgical procedure

To ensure that the surgical technique in the experimental arm is performed properly in all centres, a quality control assessment will be conducted as follows. A video vignette of the surgical procedure which has been published will be shared with all participating centres as an example^18^. The participating centres will be asked to provide a photo of the resected specimen from all included patients. Each centre will provide one or two videos of the surgical procedure when requested.

#### Postoperative

##### Assessment of endoscopic recurrence

At week 26 (6 months), an ileocolonoscopy with biopsy will be performed, and the modified Rutgeerts score determined, according to current standard guidelines^19^. The procedure will be video recorded for blinded central reading. If a video recording of the endoscopy is not available, photos taken during endoscopy will be evaluated by the same blinded central reading team. A minimum of five photos of the terminal ileum and five photos of the anastomosis are required.

##### Postoperative morbidity

Data on postoperative morbidity will be collected. Adverse events (AE) and severe adverse events (SAE) will be recorded during hospital stay and outpatient visits.

##### Assessment of clinical recurrence

The CDAI score will be determined at 6 and 12 months postoperatively, based on 7-day scoring by the patient.

##### Assessment of quality of life

QoL will be assessed up to 1 year postoperatively. QoL questionnaires will be administered at each visit: EuroQol-5 Dimension (EQ-5D) questionnaire, SF-36, and IBDQ. Patients will complete the questionnaires at baseline, 6 months, and 12 months postoperatively.

##### Medical management

In both arms, patients will be advised to stop all medications postoperatively. If a patient is at high risk of developing clinical recurrence within 6 months after surgery (that is, more than two risk factors according to ECCO guidelines), the patient may receive prophylactic medical treatment postoperatively, at the discretion of the local multidisciplinary team. If clinical recurrence occurs within 6 months, this will have to be confirmed by endoscopy, after which medical treatment can be initiated. Medications can be initiated based on the severity of the endoscopic recurrence and according to the treating physician’s preference.

During follow-up, data will be collected on the medical management of CD to determine the timing of resuming immunosuppressive medications (for example, corticosteroids, immunomodulators, and biologics).

##### Assessment of surgical recurrence

Patients will be followed up for up to 5 years after surgery to determine the reoperation rate for recurrence of disease at the anastomotic site.

### Sample size calculation

The primary endpoint is postoperative endoscopic recurrence of CD at 6 months. This study is powered to detect a difference of 25 per cent in endoscopic recurrence at 6 months between the two randomized surgical techniques, 60 per cent *versus* 35 per cent (a risk reduction of 45 per cent). Assuming a test of two independent proportions powered at 80 per cent, an α-level of 0.05, a total of 62 patients in each surgical arm will be required, for a total enrolment of 124 patients. Allowing for a 10 per cent dropout, the aim is therefore to enrol 138 patients.

### Statistical analysis

All data will be collected into an electronic database, which is the electronic data management system Castor EDC Version 1.4 (https://www.castoredc.com). Outcome parameters will be analysed with appropriate statistical tests by a statistician blinded to treatment allocation on an intention-to-treat basis using the statistical program SPSS^®^ (IBM, Armonk, New York, USA). Descriptive statistics will be used to report baseline patient and surgical variables. The primary outcome of endoscopic recurrence at 6 months following ICR, and secondary outcomes, including postoperative morbidity, clinical recurrence, and need for immunosuppressive medication, in the two surgical arms will be analysed using the chi-square or Fisher’s exact test. Differences in QoL will be analysed using mixed-model analysis of variance for repeated measures. Univariate associations with the risk of recurrence will be assessed using the Cox proportional hazards model, with results reported as hazard ratios (HRs) and 95 per cent confidence intervals. The α-level will be set at 0.050 for statistical significance.

Multiple variable models will be considered in the same way, depending on the number of recurrences identified. The most recent version of the statistical program IBM SPSS Statistics for Windows will be used.

The statistical analysis plan will be finalized before data are locked for analysis, and a decision will be made on planned subgroup analysis and how to deal with protocol violations and potential baseline imbalance.

### Safety reporting

This study is considered a low-risk trial, in which two well known and commonly performed standard treatment approaches that are currently used for CD or colon carcinoma will be compared. All adverse and serious adverse events will be monitored until they have abated or until a stable situation has been reached. Depending on the event, follow-up may require additional tests or medical procedures as indicated, and/or referral to a general physician or a medical specialist. All (serious) adverse events will be reported for both study arms.

### Data handling and storage

Every randomized patient will be assigned a three-digit study number. Communication occurs only with this number. The full name and birth date of the patient will only be recorded on the informed consent form. Data will be digitally collected using the electronic data management system Castor EDC Version 1.4 (https://www.castoredc.com). A study coordinator will coordinate the study, monitor patient inclusion and protocol steps, data collection, and data entry, prepare and perform analyses, and report the data. Continuous data monitoring will guarantee complete and real-time prospective recording of data. Local investigators will send all data (personal, medical, and other relevant information) to the AMC.

### Public disclosure and publication policy

The SPICY trial is registered at ClinicalTrials.gov, registration number NCT04538638. The results of the SPICY trial will be submitted to a peer-reviewed journal, regardless of study outcomes. Co-authorship will be based on the International Committee of Medical Journal Editors (ICMJE) guidelines.

## Discussion

Over the past few decades, treatment strategies for ileocolic CD have improved with novel medical therapies, improvement of surgical techniques, treat-to-target, therapeutic drug monitoring concepts, and close surveillance. However, all approaches have focussed on the affected bowel. The hypertrophic adipose tissue surrounding the affected ileum is a hallmark of CD and correlates with sites of severe inflammation. Despite early descriptions of the role of the mesentery in CD since 1932^20^^,^^21^, in routine clinical practice, this tissue has been largely ignored for decades. Surgical guidelines emphasize the importance of sparing as much tissue as possible (bowel and mesentery), and in routine clinical practice, a limited close bowel resection is performed in CD patients undergoing surgical resection^15^^,^^22^. The aim of the trial is to prospectively determine the postoperative endoscopic recurrence rates following a mesenteric sparing resection *versus* a more extensive mesenteric resection, when performing an ICR for CD.

It is suggested that the mesentery is an active participant in CD. Transmural inflammation in CD facilitates increased bacterial translocation into the adjacent mesentery. These translocating antigens activate adipocytes, which are cells that have complex metabolic and immunologic functions^23^. Additionally, it is thought that functional abnormalities in the mesenteric structures exert an inflammatory effect—secretion of adipokines, neuropeptides with endocrine functions, contributes to immunomodulation. Furthermore, the lymphatics in the mesentery may obstruct, remodel, and impair contraction, contributing to the irregularly thickened mesentery seen in CD. Interestingly, interaction between adipokines, neuropeptides, and lymphatic endothelia leads to adipose tissue remodelling^13^. The authors have previously demonstrated that the presence of pro-inflammatory macrophages in the mesentery is a reliable parameter for active CD, and performing total mesorectal excision in CD patients undergoing proctectomy does result in improved postoperative outcomes^9^^,^^10^. In addition, to provide a biological rationale for this trial, the authors previously analysed the mesenteric immune cells from CD patients undergoing ICR, demonstrating a pro-inflammatory phenotype in the central mesenteric area in these patients^24^.

There are several fundamental theories on the role of the mesentery contributing, via inflammatory pathways, the adipocytes or microbiome which should be addressed in future research.

In this trial, two well known and commonly performed standard surgical treatment approaches that are currently used for CD or colon carcinoma will be compared. Changing the technique into a more extensive mesenteric resection should not be at the expense of more extensive bowel resection. Some have suggested an oncological resection with more extensive mesenterectomy and ligation of the ileocolic trunk (NCT04573892, NCT04539665, NCT03769922). However, taking the ileocolic vessel at its base (proximal to the ascending branch of the ileocolic artery) might lead to a more extensive resection on the colonic side, which might influence postoperative outcomes. It also has the potential risk of impacting on vascularization of the bowel ends being anastomosed. Alongside this trial, a technique for a more extensive mesenteric resection is proposed, wherein the ileocolic trunk is preserved and transection on the colonic side is similar to the close bowel ICR (*Figs 1* and *2*)^18^.

In the authors’ opinion, there are currently two questions regarding optimizing surgical treatment for ileocolic CD: which type of anastomosis gives the best outcomes (side-to-side *versus* end-to-end *versus* Kono-S) and what the role of the mesentery is. As there is no consensus on the first question, the authors have decided not to include the Kono-S anastomosis in this study. As both techniques (excision of the mesentery or excluding the mesentery from the anastomosis via the Kono-S approach) are considered to have a significant impact on postoperative recurrence rates, the authors have decided that both questions should be answered in separate studies. The Kono-S anastomosis could potentially be a confounding factor. Therefore, participating centres will not perform a Kono-S anastomosis in patients consenting to this study.

The primary outcome in this study is the endoscopic recurrence rate. Previous studies analysing outcome following ileocaecal resection generally used surgical recurrence rate as the primary outcome. The authors have chosen not to use this outcome, as first, interpretation of surgical recurrence is influenced by patient and doctors’ preferences, with alternatives to re-resection (balloon dilatation or stricturoplasty in cases of stenosis and (new) medical treatment in cases of inflammatory recurrence). Second, a substantial proportion of re-resections are related to postoperative complications, resulting in anastomotic stricture without disease activity. Third, there has been a trend in reductions in surgical recurrence over the past decades, leading to low incidence^3^^,^^25^^,^^26^. In the long-term follow-up of the LIR!C trial, there were no surgical recurrences seen after a median follow-up of 5.2 years^3^. As endoscopic recurrence almost always precedes clinical recurrence, and can be determined by central reading performed by a gastroenterologist blinded to the intervention, this was considered a more robust primary outcome.

Analysing these two treatment strategies in a head-to-head comparison will allow an objective evaluation of the clinical relevance of mesenteric resection in CD. If a clinical benefit can be demonstrated, this could result in changes to current guidelines.

## Collaborators

Willem A. Bemelman (Amsterdam UMC, Location AMC, Amsterdam, the Netherlands); Christianne J. Buskens (Amsterdam UMC, Location AMC, Amsterdam, the Netherlands); Geert R. A. M. D’Haens (Amsterdam UMC, Location AMC, Amsterdam, the Netherlands); André. D’Hoore (University Clinics Gasthuisberg, Leuven, Belgium); Silvio Danese (Humanitas University, Milan, Italy; IRCCS Humanitas Research Hospital, Milan, Italy); Marjolijn Duijvestein (Amsterdam UMC, Location AMC, Amsterdam, the Netherlands); Krisztina B. Gecse (Amsterdam UMC, Location AMC, Amsterdam, the Netherlands); Roel Hompes (Amsterdam UMC, Location AMC, Amsterdam, the Netherlands); Bart G.P. Koot (Emma Children’s Hospital, Amsterdam UMC, Location AMC, Amsterdam, the Netherlands); Fleur Indemans (Maasziekenhuis Pantein, Beugen, the Netherlands); Amy L. Lightner (Cleveland Clinic Foundation, Cleveland, Ohio, USA); Marco W. Mundt (Flevo Hospital, Almere, the Netherlands); Antonino Spinelli (Humanitas University, Milan, Italy; IRCCS Humanitas Research Hospital, Milan, Italy); Jarmila D. W. van der Bilt (Flevo Hospital, Almere, the Netherlands); Koen W. A. van Dongen (Maasziekenhuis Pantein, Beugen, the Netherlands); Séverine Vermeire (University Clinic Gasthuisberg, Leuven, Belgium); and Sander Zwaveling (Emma Children’s Hospital, Amsterdam UMC, Location AMC, Amsterdam, the Netherlands).

## Funding

The SPICY trial is an investigator study, with funding from TKI-LSH and third-party funding from Stryker European Operations B.V., with no influence on protocol writing and no access to data.
